# Organophosphate Pesticide Exposure and Attention in Young Mexican-American Children: The CHAMACOS Study

**DOI:** 10.1289/ehp.1002056

**Published:** 2010-08-19

**Authors:** Amy R. Marks, Kim Harley, Asa Bradman, Katherine Kogut, Dana Boyd Barr, Caroline Johnson, Norma Calderon, Brenda Eskenazi

**Affiliations:** 1 Center for Children’s Environmental Health Research, School of Public Health, University of California, Berkeley, Berkeley, California, USA;; 2 Emory University, Rollins School of Public Health, Atlanta, Georgia, USA;; 3 Center for the Health Assessment of Mothers and Children of Salinas, Clinica de Salud del Valle de Salinas, Salinas, California, USA

**Keywords:** ADHD, attention, Child Behavior Checklist, DAPs, farmworker, Mexican Americans, neurobehavior, organophosphates, pesticides

## Abstract

**Background:**

Exposure to organophosphate (OP) pesticides, well-known neurotoxicants, has been associated with neurobehavioral deficits in children.

**Objectives:**

We investigated whether OP exposure, as measured by urinary dialkyl phosphate (DAP) metabolites in pregnant women and their children, was associated with attention-related outcomes among Mexican-American children living in an agricultural region of California.

**Methods:**

Children were assessed at ages 3.5 years (*n* = 331) and 5 years (*n* = 323). Mothers completed the Child Behavior Checklist (CBCL). We administered the NEPSY-II visual attention subtest to children at 3.5 years and Conners’ Kiddie Continuous Performance Test (K-CPT) at 5 years. The K-CPT yielded a standardized attention deficit/hyperactivity disorder (ADHD) Confidence Index score. Psychometricians scored behavior of the 5-year-olds during testing using the Hillside Behavior Rating Scale.

**Results:**

Prenatal DAPs (nanomoles per liter) were nonsignificantly associated with maternal report of attention problems and ADHD at age 3.5 years but were significantly related at age 5 years [CBCL attention problems: β = 0.7 points; 95% confidence interval (CI), 0.2–1.2; ADHD: β = 1.3; 95% CI, 0.4–2.1]. Prenatal DAPs were associated with scores on the K-CPT ADHD Confidence Index > 70th percentile [odds ratio (OR) = 5.1; 95% CI, 1.7–15.7] and with a composite ADHD indicator of the various measures (OR = 3.5; 95% CI, 1.1–10.7). Some outcomes exhibited evidence of effect modification by sex, with associations found only among boys. There was also limited evidence of associations between child DAPs and attention.

**Conclusions:**

*In utero* DAPs and, to a lesser extent, postnatal DAPs were associated adversely with attention as assessed by maternal report, psychometrician observation, and direct assessment. These associations were somewhat stronger at 5 years than at 3.5 years and were stronger in boys.

Organophosphate (OP) pesticides are potent toxicants that target the nervous systems of insects and other pests (Jokanovic 2009). OPs operate primarily through the inhibition of acetylcholinesterase, an enzyme that degrades the neurotransmitter acetylcholine, resulting in a buildup of acetylcholine in the neuronal junction (Jeyaratnam and Maroni 1994). However, cholinergic inhibition may not be the sole mechanism of effect, particularly in cases of low-level exposure (Costa 2006; Ray and Richards 2001; Timofeeva et al. 2008). Although accounts of human poisoning with these compounds have reported symptoms such as impaired concentration, slowed information processing and motor function, anxiety, confusion, tremors, seizure, and death (Jeyaratnam and Maroni 1994; Levin and Rodnitzky 1976), few studies have investigated the health effects in humans of low-level chronic exposure to OP pesticides. The potential effects of such low-level exposures on neurobehavioral functioning are particularly relevant for fetuses and young children, who may be especially vulnerable to neurotoxicants because of their immature nervous systems, their rapid rate of brain growth and development *in utero* and during early childhood, and their low levels of the enzymes involved in the metabolism and detoxification of OP pesticides (Eskenazi et al. 1999; Huen et al. 2009). Animal studies have found adverse effects of OP exposure during the prenatal and early postnatal periods (Costa 2006; Eskenazi et al. 1999).

Although research is limited, a few recent epidemiologic studies have reported associations between *in utero* OP exposure and adverse effects on neurobehavioral development (Engel et al. 2007; Eskenazi et al. 2007; Rauh et al. 2006; Young et al. 2005). These studies have found that biomarkers of prenatal OP exposure are associated with an increased number of abnormal neonatal reflexes, as measured by the Brazelton Scales of Neonatal Development (Engel et al. 2007; Young et al. 2005), and poorer mental development in early childhood, as measured on the Bayley Scales of Infant Development (Eskenazi et al. 2007; Rauh et al. 2006). Rauh et al. (2006) also found in a cohort of 3-year-olds living in New York City that concentrations of the OP pesticide chlorpyrifos in maternal serum were associated with the mothers’ report of symptoms consistent with pervasive developmental disorder (PDD) as well as of attention deficit/hyperactivity disorder (ADHD) or attention problems. A recent cross-sectional study also reported associations between child OP metabolite concentrations and ADHD in 8- to 15-year-olds representative of the U.S. population (Bouchard et al. 2010). Although we previously reported that prenatal OP metabolite concentrations were associated with mothers’ report of PDD symptoms in 2-year-olds living in the agricultural Salinas Valley of California, we did not find an association between maternal or child dialkyl phosphates (DAPs) and measures of attention at 2 years of age (Eskenazi et al. 2007). However, the children in our study were younger—potentially too young at 2 years of age to manifest attention problems or for mothers to detect them. Although ADHD, characterized by inattention, impulsivity, and/or hyperactivity, is occasionally diagnosed in children as young as 2 or 3 years, it is more apparent after children begin school and is more commonly reported in boys than in girls (Pastor and Reuben 2008; Staller and Faraone 2006).

The aim of this paper is to investigate associations between *in utero* and childhood OP pesticide exposure as assessed by urinary DAP metabolites and attention-related outcomes in 3.5- and 5-year-old Mexican-American children living in an agricultural community in California. We include assessment of attentional problems based not only on maternal report, as in previous studies, but also by direct neuropsychological testing and psychometrician observation.

## Materials and Methods

### Participants and recruitment

The Center for the Health Assessment of Mothers and Children of Salinas (CHAMACOS) is a prospective birth cohort aimed at studying the association of pesticides and other environmental agents on the health of pregnant women and their children living in the Salinas Valley, California. Details for this study have been described previously (Eskenazi et al. 2004, 2007). Briefly, women in their first half of pregnancy were recruited between October 1999 and October 2000 from participating prenatal care clinics serving the farmworker community. Eligible women were ≥ 18 years of age, eligible for California’s low-income health insurance program, planning to deliver at the county medical center, and spoke Spanish or English. Women gave written informed consent to participate. The study was approved by the institutional review board at University of California, Berkeley.

We followed 526 women to delivery of a liveborn, surviving singleton. We excluded from the present analyses children who did not have a prenatal DAP metabolite measurement (*n* = 2), who had a medical condition that could affect neurobehavioral assessment (*n* = 3; deafness, Down syndrome, hydrocephalus), or who were lost to follow-up or did not participate at the 3.5- or 5-year study visit (*n* = 173). The final population included 348 children who had available data at 3.5 and/or 5 years of age. Total DAPs were measured in maternal urine collected at two time points during pregnancy and in child urine collected at the 3.5-year and 5-year visits. Levels of DAPs were similar in mothers of children included in the present analysis relative to those whose children were not (*t*-test *p* = 0.40). Distributions of other covariates measured before 3.5 years of age were also similar, except that more boys than girls had dropped from the study (χ^2^ *p* = 0.04).

### Maternal interviews and assessments

We interviewed mothers twice during pregnancy (mean gestation, 14.0 and 26.6 weeks), after delivery, and when children were 6 months and 1, 2, 3.5, and 5 years of age. Interviews were conducted by bilingual, bicultural interviewers in Spanish or English. Mothers were administered the Peabody Picture Vocabulary Test (PPVT) (Dunn and Dunn 1981; Dunn et al. 1986) at the 6-month visit to assess receptive vocabulary, which was used as an indicator of verbal intelligence, and the Center for Epidemiologic Studies Depression Scale (CES-D) (Radloff 1977) at the 3.5-year visit. A registered nurse abstracted prenatal and delivery medical records.

### Attention-related outcomes

We measured attention-related outcomes in three ways: maternal report of child behavior at 3.5 and 5 years of age; direct assessment of the child at 3.5 and 5 years; and psychometrician’s report of the behavior of the child during testing at 5 years.

Mothers completed the Child Behavior Checklist (CBCL) for 1.5–5 years of age (Achenbach and Rescorla 2000) as part of the maternal interview at the 3.5- and 5-year visits to assess emotional/behavioral problems and competencies of the children. This 99-item scale, which is available in English and Spanish and has been widely used in cross-cultural research, collects data on a variety of behaviors that the parent rates as “not true,” “somewhat true,” or “very true/often true” currently or within the preceding 2 months. Questions are combined to create scores reflecting possible problem areas, including some corresponding to *Diagnostic and Statistical Manual of Mental Disorders*, 4th ed. (DSM-IV) diagnoses (American Psychiatric Association 2000) and others reflecting problem syndrome areas. The present analyses focused on two related attention scores: the attention problems scale and the DSM-related ADHD scale. Scores can be examined continuously or by the proportion above a standard cutoff score, for example, > 93rd percentile (borderline clinical range) or > 97th percentile (clinical range). Because few children fell into the clinical-range category for these scales (*n* = 3–11), we used the borderline clinical range cut-point as well as continuous raw scores in multivariate analyses.

Children also completed a battery of neurodevelopmental tests at each visit. At the 3.5-year visit, the visual attention subtest of the NEPSY-II (Korkman et al. 1997) was administered to children by trained psychometricians. Children were asked to circle specific images on a page of pictures that included distracters. For data analysis, we used continuous scores scaled to a normative sample of U.S. children; the age-standardized mean ± SD for this subtest is 10 ± 3.

During the 5-year neurodevelopmental assessment, which took approximately 2 hr to complete, trained psychometricians administered to children the Conners’ Kiddie Continuous Performance Test (K-CPT) (Conners and Staff 2001). The K-CPT is a 7-min computerized test that assesses reaction time, accuracy, and impulse control. Briefly, children were instructed to press the space bar when they saw any image on the computer screen except a ball. The computer program yields *T*-scores age-standardized to a general U.S. population (mean ± SD = 50 ± 10) for errors of commission (i.e., the child responds when he or she should not), errors of omission (i.e., the child fails to respond when he or she should), and hit reaction time. We examined *T*-scores continuously and categorically using the cutoff of *T*-score > 65, which is considered markedly atypical. The program also combines measures to generate a clinical ADHD Confidence Index score (range, 0–100). For statistical analyses, we use continuous Confidence Index percentiles and selected a cut-point of > 70th percentile (meaning that 70% of children performing similarly on the test could be correctly classified as having clinical ADHD).

Following the 5-year neurodevelopmental assessment, psychometricians blinded to exposure status answered several subjective questions evaluating the behavior of the child during the 2-hr visit, including four questions derived from the seven-item Hillside Behavior Rating Scale (Gittelman and Klein 1985). We summed responses to two questions assessing motor activity and distractibility to create an adapted ADHD symptoms scale. The Hillside Scale is associated with parent and teacher ratings and has been found to add significantly to the clinical prediction of ADHD (Willcutt et al. 1999). We created a dichotomized Hillside outcome variable with scores ≥ 7 of 12 possible points (representing < 10% of children) to flag children displaying a higher degree of attention problems based on psychometricians’ observation.

To identify children whose behaviors on multiple indices at 5 years of age were most suggestive of possible ADHD within our cohort, we created a composite ADHD variable that combined maternal report (CBCL), child testing (K-CPT), and psychometrician report (Hillside). Children were coded 1 for the ADHD indicator if they met at least two of the following conditions at 5 years of age: CBCL ADHD scale in borderline clinical range (maternal report); standardized K-CPT ADHD Confidence Index ≥ 60% (child testing); and Hillside ADHD scale in the upper quartile of values (psychometrician report).

The CBCL was completed by 331 mothers at the 3.5-year interview and 323 mothers at the 5-year interview. NEPSY scores were available for 320 3.5-year-olds, and K-CPT scores were available for 312 5-year-olds. Hillside scores were available for 322 5-year-olds.

### Pesticide exposure measurement

Details of urine collection, analysis, and quality control procedures, including detection limits and use of blanks and spikes, are described elsewhere (Bradman et al. 2005). Urine specimens were typically collected at the time of interview/neurodevelopmental assessment and were aliquoted and stored at −80°C until shipment on dry ice to the Centers for Disease Control and Prevention (CDC) for analysis. DAPs were measured using gas chromatography-tandem mass spectrometry and quantified using isotope dilution calibration (Bravo et al. 2002).

We measured six DAP metabolites in maternal and child urine: *a*) three dimethyl phosphate (DM) metabolites (dimethylphosphate, dimethylthiophosphate, dimethyldithiophosphate), representing breakdown of *O,O*-dimethyl-substituted OP pesticides such as malathion, oxydemeton-methyl, and dimethoate, and *b*) three diethyl phosphate (DE) metabolites (diethylphosphate, diethylthiophosphate, and diethyldithiophosphate) derived from *O,O*-diethyl-substituted OP pesticides such as chlorpyrifos and diazinon (Bradman et al. 2005). These six metabolites cannot be traced back to individual pesticides but, taken together, represent the breakdown products of approximately 80% of the total OP pesticides used in the Salinas Valley. Values below the limit of detection (LOD) were assigned a value of 
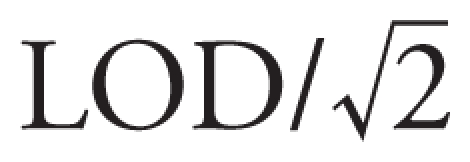
. Urinary creatinine concentrations were determined using a commercially available diagnostic enzyme method (Vitros CREA slides; Ortho Clinical Diagnostics, Raritan, NJ).

Lead, which we considered a potential confounder, was measured in cord blood by the California State Department of Public Health (Sacramento, CA) and in children’s blood at age 2 years by the Monterey County Public Health Laboratory (Salinas, CA) using graphite furnace atomic absorption spectrophotometry. Lead levels were abstracted from laboratory and clinic medical records by the study staff.

### Data analysis

We performed analyses using Stata version 10.1 (StataCorp, College Station, TX).

DAPs (nanomoles per liter) were summed and transformed to the log (base 10) scale; coefficients and odds ratios (ORs) thus represent the change in mean behavioral scores or relative odds of an outcome for each 10-fold increase in DAP concentration.

Pregnancy DM, DE, and total DAP values were created by averaging the two log-transformed pregnancy measures. For 19 women with only one DAP measurement in pregnancy, the single measure was used. Several children were missing DAP measures at 3.5 years (*n* = 58) and 5 years (*n* = 14). Separate models were run with maternal DAPs only, child DAPs only, and the two together in the same model.

We constructed separate linear regression models for continuous outcomes (e.g., NEPSY Visual Attention, CBCL scale scores) and logistic regression models for categorical outcomes (e.g., borderline clinical-range CBCL scores, markedly atypical K-CPT scores). Values of OR > 1.0 or β > 0.0 indicate positive associations between DAP levels and increased risk of adverse outcome, except for the NEPSY visual attention subtest, where a β > 0.0 indicates better performance. *p*-Values < 0.05 were considered statistically significant for tests of main effects. Model diagnostics were performed and found acceptable.

We included the same covariates in all models, except that a variable for psychometrician was not included for the CBCL measures. Covariates were selected for these analyses if they were related to conditions of testing or related to neurobehavior in the literature and/or associated with more than one outcome (*p* < 0.10 or changes to the main effect coefficient by ≥ 10%). We included in the models psychometrician (*n* = 2 at 3.5 years, *n* = 4 at 5 years), exact age at assessment, sex, maternal education, depressive symptoms, PPVT (continuous), ≥ 15 hr out-of-home child care/week, and breast feeding duration (months). In addition to these covariates, we examined the potential confounding effects of a number of other variables that did not markedly alter the observed associations and were therefore not used in models, including maternal age, parity, marital status, active/passive smoking exposure and regular alcohol use during pregnancy, and presence of father in home, maternal work status, and household income at the time of assessment. Household income above or below poverty was determined by comparing total household income with the federal poverty threshold for a household of that size (U.S. Census Bureau 2000). Covariates in final models were categorized as noted in Table 1, unless otherwise specified above. Maternal depression was missing for 5% of observations and maternal PPVT for < 1%. Values of maternal depression measured when the child was 1 year old were available and substituted for 13 instances where maternal depression when the child was 3.5 years was missing. To preserve the size of the analytic population, remaining missing covariate values (six instances for maternal depression and three for maternal PPVT) were imputed by simple random selection of a value from participants with nonmissing values.

We conducted a number of sensitivity analyses. We re-ran all models using creatinine-adjusted DAP metabolite concentrations. In addition, we added to final models some factors potentially on the causal pathway (i.e., birth weight, gestational duration). We also considered whether controlling for lead (log_10_-transformed), a known neurotoxicant, altered results for DAP concentrations in the subsamples with cord (*n* = 229) or 2-year (*n* = 296) lead values. Finally, because rates of ADHD vary considerably by sex in the general population (Pastor and Reuben 2008) and effects of other toxicants have been found to vary by sex (Delaney-Black et al. 2004), we tested for interactions between DAP concentrations and child sex, using *p* < 0.15 for the interaction term to determine whether associations of DAP concentrations with measures of attention differed for boys and girls.

## Results

### Demographic characteristics

Demographic characteristics of the study population are presented in Table 1. Mean (± SD) maternal age was 26.5 ± 5.2 years at delivery. Most mothers were married or living as married (82%), spoke only Spanish at home (90%), were born in Mexico (86%), had not completed high school (79%), were nonsmokers (96%), and lived in a low-income household (97% within 200% of the federal threshold for poverty; 63% within 100%). One third of mothers were primiparous, and almost half of mothers had symptoms of depression (CES-D ≥ 16). About half of the children were in regular out-of-home child care at 3.5 years of age, and by 5 years, 69% had attended some preschool. At the time of their 5-year assessment, almost one third of children had started kindergarten.

### Urinary DAP concentrations

OP metabolite concentrations are summarized in Table 2. The geometric mean (GM) of average prenatal maternal urinary DAP concentrations was 109.0 nmol/L. The two pregnancy DAP measurements were weakly correlated (*r* = 0.15; *p* = 0.009). The DAP concentrations were lower in children than in mothers during pregnancy. Child DAPs were uncorrelated with maternal DAPs (Pearson *r* = 0.03; *p* = 0.60 with 3.5-year measures and *r* = −0.02; *p* = 0.77 with 5-year measures).

### Attention in children 3.5 and 5 years of age

When children were 3.5 years old, approximately 5% of mothers reported child behaviors that were in the borderline clinical range for attention problems and ADHD symptoms, with poorer scores on CBCL attention-related scales among boys than among girls [see Supplemental Material, Table 1 (doi:10.1289/ehp.1002056)]. The NEPSY visual attention scores averaged 8.8 ± 2.3.

When children were 5 years old, 4% of their mothers reported behaviors consistent with attention problems and 7% reported ADHD symptoms in the borderline clinical range; again, scores were higher in boys than in girls [see Supplemental Material, Table 1 (doi:10.1289/ehp.1002056)]. On the K-CPT, 19% of children scored markedly atypical for errors of omission, 17% for errors of commission, and 6% for hit reaction time; taken together, 8% of children scored ≥ 70% on the standardized ADHD Confidence Index scale. Boys had higher average scores on the ADHD Confidence Index than girls [see Supplemental Material, Table 1 (doi:10.1289/ehp.1002056)]. Psychometricians rated 7% of children ≥ 7 on ADHD-related behaviors on the Hillside scale, and 8.5% of children were classified as having ADHD symptoms at 5 years according to the composite score.

Continuous CBCL scores were correlated across ages (Pearson *r* = 0.46–0.54; *p* < 0.01), and at each age, all continuous attention measures were correlated with one another (Pearson *r* = 0.16–0.85; *p* < 0.01) except for the NEPSY test of visual attention, which was not correlated with any outcome (*r* = −0.09 to 0.04).

### Relation between prenatal DAPs and attention

Table 3 (categorical outcomes) and Table 4 (continuous outcomes) present the relationship of prenatal DAP concentrations and measures of child attention, not controlling for child DAPs in the models. Adding child DAPs as a covariate produced very similar results (data not shown).

When the children were 3.5 years of age, prenatal DAP concentrations were positively associated with attention problems [OR = 3.0; 95% confidence interval (CI), 0.7–11.7; *p* = 0.12] and ADHD (OR = 3.1; 95% CI, 0.8–11.5; *p* = 0.09) in the borderline clinical range on the CBCL, although estimates were not statistically significant.

When the children were 5 years of age, prenatal total DAP levels, and DM levels specifically, were associated with maternal report (CBCL) of both ADHD scores and poorer attention scores when the scores were examined as continuous outcomes (Table 4) but not as categorical outcomes (Table 3). Total DAP concentrations (and total DM and DE concentrations separately) were nonsignificantly associated with the continuous K-CPT ADHD Confidence Index scores (Table 4) and were significantly associated with this index when it was modeled as a dichotomous variable (Table 3). Specifically, for each 10-fold increase in DAP concentrations, children had five times the odds of scoring > 70% on the ADHD Confidence Index (OR = 5.1; 95% CI, 1.7–15.7). Prenatal total DAP concentrations were nonsignificantly associated with the psychometrician Hillside ratings of poor attention (OR = 3.0; 95% CI, 0.9–9.8; *p* = 0.06). Total DAP concentrations, and DM concentrations specifically, were associated with having ADHD signs using the composite indicator (total DAPs OR = 3.5; 95% CI, 1.1–10.7), which is based on the three assessment methods (i.e., ADHD signs based on least two of the three following measures: maternal report on the CBCL; child performance on the K-CPT; and psychometrician ratings on the Hillside scale) and is thus not independent of the individual components.

Using creatinine-adjusted DAP levels led to conclusions similar to those presented above [Supplemental Material, Tables 2 and 3 (doi:10.1289/ehp.1002056)]. Some relationships were stronger, whereas others were attenuated. In addition, birth weight, gestational age, breast-feeding, and lead did not confound associations between DAPs and attention or ADHD, because these factors did not change the main effect estimates by > 10% when added to or removed from models.

We also tested whether the associations of prenatal DAP concentrations and measures of attention were different for boys and girls, as shown in Table 5 (categorical outcomes) and Table 6 (continuous outcomes). We found evidence for effect modification by sex on the association of prenatal DAP metabolite concentrations and maternal report of attention problems and ADHD symptoms on the CBCL; at 3.5 years and 5 years, no associations were seen for girls, but among boys, DAP concentrations were significantly associated with poorer attention and ADHD scores (Table 6). We also observed stronger associations among boys than among girls for several categorical outcomes, including CBCL outcomes in the borderline clinical range, high K-CPT Confidence Index scores, scoring ≥ 7 on the Hillside ADHD scale, and the composite measure, but only the interaction terms for the Hillside ADHD scale and composite ADHD indicator models were statistically significant (*p* < 0.15) (Table 5).

### Relation between child DAPs and attention

We did not observe statistically significant associations between children’s concurrent total DAP concentrations and any of the measures of attention, whether we adjusted for creatinine or not (Tables 7 and 8) and with or without (not shown) maternal DAPs in the models. However, we did observe that for every 10-fold increase in child urinary DE concentration at 5 years, there was a doubling in the odds in the ADHD composite indicator variable (OR = 2.0; 95% CI, 1.1–3.6). We observed no statistically significant increase in adverse outcomes with child DM concentrations.

## Discussion

We found that higher concentrations of OP metabolites in the urine of pregnant women were associated with increased odds of attention problems and poorer attention scores in their young children. This finding was relatively consistent using different approaches to assess attentional difficulties, for example, by maternal report, psychometrician observation, or neuropsychological testing. These associations appeared to be somewhat stronger at 5 years than at 3.5 years and were generally stronger in boys than in girls. Children’s concurrent total DAP and DM metabolite levels at 3.5 years and 5 years were unrelated to attention outcomes, but we observed some evidence that child DE concentrations at 5 years were adversely related to our composite measure of attention.

Although OP pesticides are designed to target the nervous systems of pests, few studies have investigated a possible role for exposure to these chemicals on children’s neurobehavior. Our findings suggest that exposure to OP pesticides may affect the attention of young children. Although we did not observe similar associations in this cohort between prenatal DAP concentrations and attention on the CBCL among 2-year-olds (Eskenazi et al. 2007), such behaviors may not yet have been evident or considered atypical at this younger age.

Our current findings are consistent with those from a cohort study of African-American and Dominican-American children in New York City, which found prenatal blood concentrations of chlorpyrifos to be associated with maternal report of ADHD/attention problems at 3 years of age (Rauh et al. 2006) using the CBCL, the same instrument we used at 2, 3.5, and 5 years of age. Our study is also consistent with a study of a nationally representative sample that reported associations between child DAP concentrations (primarily DMs) and ADHD assessed via a parental interview (Bouchard et al. 2010). Children in that study were considerably older (8–15 years of age) than our population. Another study of Hispanic children (mean age = 7 years) living in an agricultural community reported adverse associations of child urinary DAPs and attention-related performance errors on the Wisconsin Card Sorting Test; however the population was small (*n* = 48) (Lizardi et al. 2008). In the present study, associations between attention measures and prenatal DAPs are stronger than those with child DAP concentrations. Furthermore, results with child DAPs must be viewed with caution, because they reflect concurrent exposures, the temporal relation with the outcome is unclear, and exposure may not be independent of childhood behavior.

We find some evidence of effect modification by sex of the child. The clinical presentation of attention deficit disorder may vary by the child’s sex, with girls reportedly displaying more inattentive-type problems and boys displaying more hyperactive and impulsive behaviors (Biederman et al. 2002; Staller and Faraone 2006). Studies of other substances have found marked differences in neurotoxic susceptibility by sex. For example, one study found that 6-year-old boys prenatally exposed to cocaine displayed more problem behaviors (hyperactivity) than controls but observed no such differences among girls (Delaney-Black et al. 2004). A recent meta-analysis reported that differences in brain morphology between youth with ADHD and controls varied by child sex (Hutchinson et al. 2008), and a functional imaging study found that men with ADHD displayed different neural activity patterns than controls, but women did not (Valera et al. 2010).

The mechanisms by which OPs could cause attentional problems and/or impulsivity can be only speculated, as ADHD is a complex disorder and the precise causes are unknown. However, there is considerable evidence that neurotransmitters including dopamine, noradrenaline, and serotonin, the primary target of ADHD pharmacologic treatments, are involved. Less is known about the role of the cholinergic system. Although OP pesticides at high doses will inhibit acetylcholinesterase, experimental evidence in animals suggests that even OP doses that cause no or little cholinesterase inhibition may produce biochemical and behavioral effects (Costa 2006), including adverse effects on sustained attention and an increase in impulsivity in rodents (Middlemore-Risher et al. 2010). In addition, there is a growing body of evidence that OPs may operate through a variety of noncholinergic mechanisms, such as by disruption of various cellular processes such as DNA replication and axonal and dendritic growth (Howard et al. 2005) and by oxidative stress in the developing brain (Slotkin and Seidler 2009).

Our study has many strengths. We were able to assess prenatal OP exposure and follow the children longitudinally. Our findings are generally consistent whether we use creatinine or non-creatinine-adjusted DAP metabolite concentrations. Although DAP concentrations were not associated with all outcomes, we found evidence for an association across various assessment methods, including maternal report, child testing, and subjective observation by evaluators blinded to exposure.

This study has some limitations. Notably, the assessment of exposure to OPs is challenging because of the rapid metabolism of OP pesticides (Needham 2005) and the lack of a measure of long-term exposure; however, because the prenatal measures are an average of two measurements, they may better reflect ongoing exposure during the pregnancy. Also, the prenatal maternal urinary DAP concentrations in our study are higher than in a nationally representative sample of women of childbearing age (109.0 vs. 82.3 nmol/L) (Bradman et al. 2005; CDC 2004) and therefore may not be generalizable to less-exposed populations.

Another limitation is that some of the measures we used to assess behavior in these Mexican-American children were standardized for the general U.S. population. Therefore, the percentage of children identified as having a problem may not be comparable with that expected for the general U.S. population. Nevertheless, this limitation should not affect the examination of the relative associations of DAPs and outcomes within our own homogeneous study population.

Future studies should evaluate whether associations of OP exposure and attention disorders persist in older children and in children in different populations. ADHD is often not diagnosed until children are of school age, and screening instruments may identify attention problems more accurately in older children than in preschoolers. In addition, future investigations should consider whether children with certain genetic polymorphisms (e.g., paraoxonase 1) may be more likely to show attention problems in relation to OP exposure. Approximately 8–9% of school-age children are estimated to have ADHD (Pastor and Reuben 2008); given that attention problems of children interfere with learning and social development, finding potential causes that can be remediated are of great public health importance.

## Conclusion

*In utero* DAP and, to a lesser extent, child DAP concentrations were associated adversely with attention in young children as assessed by maternal report, psychometrician observation, and/or direct assessment. These associations were somewhat stronger at 5 years than at 3.5 years and in boys than in girls.

## Figures and Tables

**Table 1:** Demographic characteristics of CHAMACOS study population, Salinas Valley, California, 2000–2001 (*n* = 348).

Characteristic	*n* (%)
Mother during pregnancy	

Parity	
0	114 (32.8)
≥ 1	234 (67.2)
Married or living as married	
Yes	285 (81.9)
No	63 (18.1)
Education	
≤ Grade 6	149 (42.8)
Grades 7–12	126 (36.2)
≥ High school graduate	73 (21.0)
Country of origin	
United States	44 (12.6)
Mexico	298 (85.6)
Other	6 (1.7)
Years in the United States	
≤ 5	162 (46.6)
6–10	90 (25.9)
≥ 11	60 (17.2)
Entire life	36 (10.3)
Smoking	
Yes	14 (4.0)
No	334 (96.0)
Depressive symptoms (CES-D)	
Yes	144 (43.8)
No	185 (56.2)
Poverty status	
At or below poverty threshold	205 (62.7)
Above poverty threshold	122 (37.3)

At child follow-up	

Child sex	
Male	163 (46.8)
Female	185 (53.2)
Child care ≥ 15 hr/week (3.5 years)	
Yes	177 (53.3)
No	155 (46.7)
Child care ≥ 15 hr/week (5 years)	
Yes	106 (32.8)
No	217 (67.2)
Any preschool by 5 years of age	
Yes	228 (69.3)
No	101 (30.7)
Kindergarten by 5 years of age	
Yes	96 (29.2)
No	233 (70.8)

**Table 2:** GM concentrations (nmol/L) of average maternal urinary DAP metabolites^a^ during pregnancy, CHAMACOS Study, Salinas Valley, California [GM (95% CI)].

Marker of exposure^a^	Pregnancy (*n* = 348)	3.5 Years (*n* = 290)	5 Years (*n* = 320)
Total DAPs	109.0 (99.4–119.6)	77.5 (65.4–91.9)	92.6 (78.6–109.0)
Total DEs	17.7 (16.1–19.4)	7.0 (5.8–8.3)	7.2 (6.0–8.7)
Total DMs	76.8 (69.3–85.0)	62.5 (52.2–74.7)	72.4 (61.0–86.0)

a Not adjusted for creatinine; measurements below the LOD were assigned a value of 
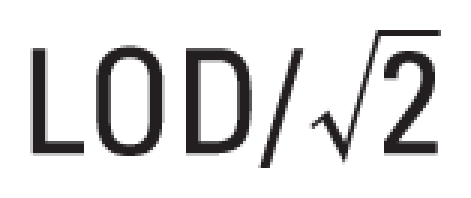

**Table 3:** Adjusted^a^ logistic models for attention-related outcomes at 3.5 and 5 years of age per 10-fold increase in prenatal urinary DAP concentrations [OR (95% CI)].

Model	*n*_outcome_/*n*	DAPs	DEs	DMs
3.5 Years				

CBCL				
Attention problems	17/330	3.0 (0.7–11.7)	2.1 (0.6–7.0)	3.2 (0.9–11.3)*
ADHD	18/329	3.1 (0.8–11.5)*	2.8 (0.9–8.9)*	1.3 (0.4–4.4)

5 Years				

CBCL				
Attention problems	13/322	0.8 (0.2–3.8)	0.7 (0.2–2.8)	2.0 (0.5–8.5)
ADHD	23/322	1.1 (0.3–3.5)	1.1 (0.4–3.2)	1.3 (0.4–4.0)
K-CPT				
Markedly atypical				
Percent omissions	59/312	1.5 (0.7–3.3)	1.3 (0.6–2.8)	1.9 (0.9–4.1)
Percent commissions	54/312	1.0 (0.5–2.2)	0.8 (0.4–1.6)	1.2 (0.6–2.7)
Hit reaction time	20/311	1.6 (0.5–5.2)	1.5 (0.5–4.6)	1.1 (0.3–3.6)
ADHD Confidence Index				
> 70th percentile	25/297	5.1 (1.7–15.7)#	3.2 (1.2–8.9)**	6.6 (2.2–19.3)#
Hillside Behavioral Rating Scale				
Attention ≥ 7 of 12	23/322	3.0 (0.9–9.8)*	2.9 (1.0–8.5)*	2.3 (0.7–7.4)
Composite ADHD indicator				
ADHD indicator	27/319	3.5 (1.1–10.7)**	3.0 (1.1–8.2)**	1.7 (0.5–5.5)

a Adjusted for psychometrician, age at assessment, sex, child care, breast-feeding, maternal education, depressive symptoms, and PPVT.

* *p* < 0.10.

** *p* < 0.05.

# *p* < 0.01.

**Table 4:** Adjusted^a^ linear models for attention-related outcomes at 3.5 and 5 years of age per 10-fold increase in prenatal urinary DAP concentrations [β (95% CI)].

Model	*n*	DAPs	DMs	DEs
3.5 Years				

CBCL				
Attention problems	330	0.3 (−0.2 to 0.7)	0.3 (−0.1 to 0.7)*	0.0 (−0.5 to 0.4)
ADHD	329	0.5 (−0.3 to 1.3)	0.6 (−0.1 to 1.3)*	−0.2 (−0.9 to 0.6)
NEPSY				
Visual attention	319	0.2 (−0.5 to 0.8)	0.1 (−0.5 to 0.6)	−0.2 (−0.8 to 0.5)

5 Years				

CBCL				
Attention problems	322	0.7 (0.2 to 1.2)**	0.6 (0.2 to 1.0)**	0.4 (−0.1 to 0.9)
ADHD	322	1.3 (0.4 to 2.1)**	1.1 (0.3 to 1.9)**	0.7 (−0.2 to 1.5)
K-CPT				
ADHD Confidence Index	297	3.4 (−1.8 to 8.7)	2.0 (−2.8 to 6.9)	3.4 (−1.7 to 8.6)

a Adjusted for psychometrician, age at assessment, sex, child care, breast-feeding, maternal education, depressive symptoms, and PPVT.

* *p* < 0.10.

** *p* < 0.05.

**Table 5:** Adjusted^a^ logistic models for attention-related outcomes at 3.5 and 5 years of age per 10-fold increase in prenatal urinary DAP concentrations, stratified by sex.

Model	Boys		Girls		*p*-Value for interaction
	*n*_outcome_/*n*	OR (95% CI)	*n*_outcome_/*n*	OR (95% CI)	
3.5 Years					

CBCL					
Attention problems	12/151	4.1 (0.8–22.2)	5/179	2.1 (0.2–29.9)	0.68
ADHD	12/151	6.4 (1.1–39.0)**	6/176	1.0 (0.1–11.2)	0.21

5 Years					

CBCL					
Attention problems	10/154	1.0 (0.2–6.0)	3/168	0.6 (0.0–17.3)	0.77
ADHD	14/154	4.9 (0.7–33.0)	9/168	0.3 (0.0–2.2)	0.18
K-CPT					
Markedly atypical					
Percent omissions	21/148	1.7 (0.4–6.4)	38/164	1.4 (0.5–4.0)	0.90
Percent commissions	24/148	0.9 (0.2–3.2)	30/164	1.2 (0.4–3.3)	0.89
Hit reaction time	7/147	1.2 (0.1–11.5)	13/164	1.7 (0.4–7.4)	0.72
ADHD Confidence Index					
> 70th percentile	14/140	10.1 (1.6– 65.3)**	11/157	3.3 (0.6–17.0)	0.41
Hillside Behavioral Rating Scale					
Attention ≥ 7 of 12	14/153	7.9 (1.4–46.0)**	9/169	1.0 (0.2–5.9)	0.14
Composite ADHD indicator					
ADHD indicator	19/150	11.1 (1.8– 66.5)#	8/169	1.1 (0.2–7.1)	0.13

a Adjusted for psychometrician, age at assessment, sex, maternal education, depressive symptoms, PPVT, child care, and breast-feeding.

** *p* < 0.05.

# *p* < 0.01.

**Table 6:** Adjusted^a^ linear models for continuous attention-related outcomes at 3.5 and 5 years of age per 10-fold increase in prenatal urinary DAP concentrations, stratified by sex.

Model	Boys		Girls		*p*-Value for interaction
	*n*	β (95% CI)	*n*	β (95% CI)	
3.5 Years					

CBCL					
Attention problems	151	0.7 (0.0 to 1.4)**	179	−0.1 (−0.7 to 0.5)	0.05
ADHD	151	1.3 (0.1 to 2.5)**	178	−0.2 (−1.2 to 0.8)	0.06
NEPSY					
Visual attention	143	0.2 (−0.8 to 1.1)	176	0.2 (−0.7 to 1.2)	0.99

5 Years					

CBCL					
Attention problems	154	0.9 (0.2 to 1.7)**	168	0.4 (−0.2 to 1.0)	0.28
ADHD	154	1.9 (0.6 to 3.2)#	168	0.6 (−0.5 to 1.6)	0.13
K-CPT					
ADHD Confidence Index	140	6.3 (−0.5 to 13.3)*	157	0.5 (−7.2 to 8.3)	0.39

a Adjusted for psychometrician, age at assessment, sex, maternal education, depressive symptoms, PPVT, child care, and breast-feeding.

* *p* < 0.10.

** *p* < 0.05.

# *p* < 0.01.

**Table 7:** Adjusted^a^ logistic regression model for attention-related outcomes at ages 3.5 and 5 years per 10-fold increase in child urinary DAP concentrations [OR (95% CI)].

Model	*n*_outcome_/*n*	Total DAPs	DMs	DEs
3.5 Years				

CBCL				
Attention problems	17/289	1.6 (0.8–3.5)	1.6 (0.8–3.3)	1.9 (0.9–3.9)
ADHD	17/288	1.4 (0.7–3.1)	1.4 (0.7–3.0)	1.0 (0.5–2.2)

5 Years				

CBCL				
Attention problems	13/319	1.0 (0.4–2.4)	0.9 (0.4–2.1)	1.8 (0.8–3.9)
ADHD	22/319	0.6 (0.3–1.2)	0.5 (0.3–1.1)*	0.9 (0.5–1.7)
K-CPT				
Markedly atypical				
Percent omissions	58/309	1.0 (0.6–1.6)	0.9 (0.6–1.5)	1.5 (1.0–2.2)*
Percent commissions	53/309	1.1 (0.7–1.7)	1.1 (0.7–1.8)	0.9 (0.6–1.4)
Hit reaction time	19/308	1.1 (0.5–2.3)	1.0 (0.5–2.0)	1.3 (0.7–2.4)
ADHD Confidence Index				
> 70th percentile	24/294	1.3 (0.7–2.5)	1.2 (0.7–2.3)	1.5 (0.8–2.8)
Hillside Behavioral Rating Scale				
Attention ≥ 7 of 12	23/319	1.4 (0.7–2.8)	1.1 (0.6–2.1)	1.4 (0.8–2.6)
Composite ADHD indicator				
ADHD indicator	25/316	1.0 (0.5–2.0)	0.8 (0.4–1.5)	2.0 (1.1–3.6)**

a Adjusted for maternal total DAPs, psychometrician, age at assessment, sex, maternal education, depressive symptoms, PPVT, child care, and breast-feeding, as well as maternal urinary DAPs.

* *p* < 0.10.

** *p* < 0.05.

**Table 8:** Adjusted^a^ linear regression models for attention-related outcomes at ages 3.5 and 5 years per 10-fold increase in child urinary DAP concentrations [β (95% CI)].

Model	*n*	Total DAPs	DMs	DEs
3.5 Years				

CBCL				
Attention problems	289	0.1 (−0.2 to 0.4)	0.1 (−0.2 to 0.3)	0.2 (0.0 to 0.5)*
ADHD	288	0.1 (−0.3 to 0.6)	0.1 (−0.3 to 0.6)	0.2 (−0.3 to 0.7)
NEPSY				
Visual attention	277	−0.1 (−0.5 to 0.3)	−0.1 (−0.5 to 0.3)	−0.1 (−0.5 to 0.3)

5 Years				

CBCL				
Attention problems	319	0.0 (−0.3 to 0.2)	−0.1 (−0.3 to 0.2)	0.0 (−0.2 to 0.3)
ADHD	319	0.0 (−0.5 to 0.5)	0.0 (−0.5 to 0.4)	0.1 (−0.3 to 0.6)
K-CPT				
ADHD Confidence Index	294	−0.7 (−3.8 to 2.3)	−1.0 (−3.9 to 1.9)	2.2 (−0.5 to 5.0)

a Adjusted for maternal total DAPs, psychometrician, age at assessment, sex, maternal education, depressive symptoms, PPVT, child care, and breast-feeding, as well as maternal urinary DAPs.

* *p* < 0.10.
